# A Trifunctional Dextran-Based Nanovaccine Targets and Activates Murine Dendritic Cells, and Induces Potent Cellular and Humoral Immune Responses *In Vivo*

**DOI:** 10.1371/journal.pone.0080904

**Published:** 2013-12-05

**Authors:** Limei Shen, Tetsuya Higuchi, Ingrid Tubbe, Nicole Voltz, Mathias Krummen, Stefanie Pektor, Evelyn Montermann, Kristin Rausch, Manfred Schmidt, Hansjörg Schild, Stephan Grabbe, Matthias Bros

**Affiliations:** 1 Department of Dermatology, University Medical Center Mainz, Mainz, Germany; 2 Department of Dermatology, Toho University Sakura Medical Center, Chiba, Japan; 3 Institute of Physical Chemistry, Johannes Gutenberg University Mainz, Mainz, Germany; 4 Institute of Immunology, University Medical Center Mainz, Mainz, Germany; University of Birmingham, United Kingdom

## Abstract

Dendritic cells (DCs) constitute an attractive target for specific delivery of nanovaccines for immunotherapeutic applications. Here we tested nano-sized dextran (DEX) particles to serve as a DC-addressing nanocarrier platform. Non-functionalized DEX particles had no immunomodulatory effect on bone marrow (BM)-derived murine DCs *in vitro*. However, when adsorbed with ovalbumine (OVA), DEX particles were efficiently engulfed by BM-DCs in a mannose receptor-dependent manner. A DEX-based nanovaccine containing OVA and lipopolysaccharide (LPS) as a DC stimulus induced strong OVA peptide-specific CD4^+^ and CD8^+^ T cell proliferation both i*n vitro* and upon systemic application in mice, as well as a robust OVA-specific humoral immune response (IgG1>IgG2a) *in vivo*. Accordingly, this nanovaccine also raised both a more pronounced delayed-type hypersensitivity response and a stronger induction of cytotoxic CD8^+^ T cells than obtained upon administration of OVA and LPS in soluble form. Therefore, DEX-based nanoparticles constitute a potent, versatile and easy to prepare nanovaccine platform for immunotherapeutic approaches.

## Introduction

Several classes of antigen-loaded particles have been demonstrated to passively target antigen presenting cells (APCs) including dendritic cells (DCs) as the most potent APC population by means of unspecific endocytotic internalization [1]. In the course of these studies, some types of particles have been demonstrated to exert immunostimulatory activity in DCs [2]. This property may be of advantage in order to evoke an antigen-specific immune response. However, a nanoparticle platform devoid of intrinsic immunomodulatory potential might be even more feasible as it allows to determine the polarization of the antigen-specific immune response solely by the quality of a particle-delivered adjuvant [3].

In this regard, we opted for dextran (DEX) nanoparticles, introduced almost thirty years ago as a carrier platform for protein antigens plus immunomodulatory compounds to elicit an antigen-specific humoral response after *in vivo* application [4]. In general, dextrans constitute dextrose-derived neutral biopolymers, which due to their excellent biocompatibility have been in widespread clinical use for decades, serving as blood volume expanders, and preventing thrombosis. Therefore, DEX particles may constitute an ideal platform for the development of functionalized nanocarriers. The DEX particles used in our study are based on commercially available dextran particles with an average Mw of 500 kDa. The model protein antigen ovalbumine (OVA), and lipopolysaccharide (LPS), a well established toll-like receptor (TLR)-4 ligand and TH1-promoting DC stimulus, were adsorbed to DEX particles by applying the protocol introduced by Schröder and co-workers [4], [5].

It has been shown that uptake of OVA by pinocytosis in DCs resulted in the activation of OVA-specific CD4^+^ T cells, but evoked no CD8^+^ T cell response [6]. In contrast, endocytotic uptake of OVA, which is efficiently bound by the mannose receptor (MR) due to its mannosylation [7], resulted in strong activation of either T cell population. The MR belongs to a group of C-type lectin receptors which act as pattern recognition receptors and bind both endogenous as well as pathogen-derived structures [8]. Due to its rather restricted expression pattern, largely confined to macrophages and myeloid DC populations, the MR has become a well established target receptor for APC-specific vaccination [9].

In this study we analyzed the efficacy of a refined MR-targeting delivery system based on OVA, intended to serve both as a MR targeting molecule and as a source of antigen. We show that DEX-based nanoparticles as such are not internalized by DCs and lack unwanted immunomodulatory function. DEX particles adsorbed with OVA, however, were efficiently engulfed by murine DCs *in vitro* in a MR-dependent manner. Codelivery of OVA and LPS by DEX particles induced stronger and more sustained immune responses *in vitro* and *in vivo* than direct application of these compounds which confirms their usability for immunotherapeutic applications.

## Materials and Methods

### Adsorption of Antigen and Adjuvant to Dextran (DEX) Particles

DEX nanoparticles were mixed with OVA and LPS according to a general protocol described to result in efficient binding of distinct compounds to dextran-based nanospheres [5] with some modifications. Detailed information are obtainable from the Supporting Informations in Methods S1.

### Electron Microscopy

Shape and size distribution of DEX particles dispersed in PBS were studied using a Tecnai 12 transmission electron microscope (FEI, Hilsboro, OR) at an accelerating voltage of 120 kV. Images were taken using a 1392×1042 SIS Megaview camera (Olympus, Münster, Germany).

### Dynamic Light Scattering

The size of DEX particles resuspended in PBS and analysis of potential interaction with blood serum were determined by dynamic light scattering (DLS) analysis as described elsewhere [10]. Detailed descriptions are given in Methods S1.

### Ethics Statement

All mouse strains were bred and maintained in the Central Animal Facilities of the University of Mainz under specific pathogen-free conditions according to the guidelines of the regional animal care committee. All animal experiments were performed in accordance with national and European (86/609/EEC) legislation, and in accordance with the Central Laboratory Animal Facility of the University Medical Center of Mainz. The protocol was approved by the national investigation office of Rhineland-Palatinate (Permit Number: 13177-07/G08-1008).

For ethical reasons, blood samples were withdrawn under ketamine and xylezine anaesthesia and all efforts were made to minimize suffering.

### Mice

All mouse strains were bred and maintained in the Central Animal Facilities of the Johannes Gutenberg-University of Mainz under specific pathogen-free conditions on a standard diet. The “Principles of Laboratory Animal Care” (NIH publication no. 85-23, revised 1985) were followed. CD4^+^ T cells of OT-II (C57BL/6 background) and of DO11.10 (BALB/c) mice are transgenic for a αβTCR specific for OVA_323–339_ peptide in context of H-2 I-A^b^ and I-A^d^, respectively. CD8^+^ T cells of OT-I (C57BL/6) mice are transgenic for a αβTCR specific for OVA-derived SIINFEKL peptide (OVA_257–264_) in the context of H-2K^b^. Both OT-I and OT-II strains (C57BL/6 background) were crossed to CD45.1^+^ C57BL/6J congenic mice.

### Generation of Murine Bone Marrow-derived Dendritic Cells

BM-DCs were generated as previously described [11] with some modifications. On day 6, non-adherent and loosely adherent BM-DCs were collected. Aliquots of BM-DCs were stimulated with DEX particle formulations at concentrations as indicated or with LPS (100 ng/ml) for 24 h.

### DC Viability

To assess potential cytotoxic effects of DEX particle formulations, day 6 BM-DCs (2.5×10^5^) were reseeded into wells of 96 well cell culture plates in a volume of 50 µl, and DEX particles were added at different concentrations as indicated. To assay cell viability, tetrazolium substrate was added which is reduced to a chromogenic formazan product by mitochondrial succinate dehydrogenase, and thereby correlates with the number of metabolically active cells. The reaction was stopped by addition of an organic solvent, and the concentration of solubilized formazan was detected spectrophotometrically in an ELISA reader according to the protocol provided by the manufacturer (Promega, Madison, WI).

### Cellular Uptake of Functionalized FITC-labeled DEX Particles

BM-DCs (5×10^5^ cells) or spleen cell suspensions (2×10^6^ cells) derived from C57BL/6 mice were incubated with FITC-labeled DEX particle formulations (each 50 µl [BM-DCs] or 30 µl [spleen cells]) as indicated in a volume of 200 µl at 37°C in 96 wells of a cell culture plate for the indicated periods of time. To assess for MR-dependent endocytosis of OVA-adsorbed DEX particles by BM-DCs, cells (5×10^5^ in 200 µl) were preincubated with mannan (200 µg/ml; Sigma-Aldrich, Deisenhofen, Germany) for 30 min at 37°C. After incubation, cells were harvested and stained for surface lineage marker expression as indicated for subsequent flow cytometry analysis (see below).

### Laser Scanning Microscopy

Day 6 BM-DCs were cocultured with FITC-labeled DEX(OVA) as described (see above) in wells of 96 well cell culture plates for the indicated periods of time. After incubation, the cells were harvested and washed with FACS buffer (PBS, 1% FCS, 0.5 mM EDTA). Subsequently the cells were transferred onto chamber slides (IBIDI, Martinsried, Germany). Cells were incubated with anti-CD11c antibody as described above, and nuclei were stained with DAPI (Life Technologies, Carlsbad, CA). Cellular uptake of DEX particle formulations was analyzed by confocal laser scanning microscopy (LSM510-UV, Zeiss, Germany).

### Flow Cytometry

Cells were washed in FACS buffer, and stained with PE-Cy7-conjugated anti-CD11c, PE-conjugated anti-CD80 or anti-CD3, FITC-conjugated-anti-CD86, APC-conjugated anti-CD19 or anti-CD40, and e-fluor405-conjugated anti-MHCII or anti-F4/80 antibodies as indicated. For intracellular detection of IFN-γ, cells were stained with APC-Cy7-conjugated CD8, PE-conjugated anti-Vα2, and PE-Cy5-conjugated CD45.1, and fixed with 4% paraformaldehyde. Then, cells were permeabilized, and stained with APC-conjugated anti-IFN-γ. All antibodies were purchased from eBioscience (San Diego, CA). Expression intensities were assessed by flow cytometry (FACS LSR II, BD Biosciences, San Diego, CA).

### T Cell Proliferation Assays

Aliquots of day 6 BM-DCs (10^6^ cells) were cocultured with DEX particle formulations (100 µl), LPS (100 ng/ml), and OVA protein (2 µg) as indicated for 24 h. For *in vitro* proliferation assays, (OT-II) T cells were purified from mouse spleens and lymph nodes by auto MACS (Miltenyi Biotec GmbH, Bergisch Gladbach, Germany). BM-DCs were cocultured with OT-II T cells at the indicated cell numbers in triplicates for 48 h. Afterwards, cocultures were incubated with ^3^H-thymidine for an additional 16 h. Genomic incorporation of ^3^H-thymidine was determined by liquid scintillation counting.

For *in vivo* analysis of T cell proliferation, splenocytes (OT-IxLy-5.1, OT-IIxLy5.1) were incubated with 0.5 µM carboxyfluorescein diacetate succinimidyl ester (CFSE, Life Technologies) for 10 min at room temperature. CFSE-labeled splenocytes (10^7^) were transferred intravenously (*i.v.*) into C57BL/6 mice. After 48 h, 4 µg of OVA protein or the corresponding amount of DEX particle-bound OVA were injected *i.v.*, either alone or combined with 100 ng of LPS as indicated. Four days later, spleens and peripheral lymph nodes (LNs) were removed and cell suspensions were analyzed for proliferation of CFSE-labeled OT-IxLy-5.1 or OT-IIxLy5.1 T cells by flow cytometry.

### Foot Pad Swelling Assay to Assess Antigen-specific Delayed-type Hypersensitivity

DO11.10 CD4^+^ T cells (5×10^6^ per mouse) were injected *i.v.* into BALB/c mice. One day later, OVA_323–339_ peptide (40 µg/mouse), LPS (100 ng/mouse), and DEX particle formulations (40 µg/mouse) were injected *i.v.* as indicated. Two weeks later, syngeneic day 7 BM-DCs, stimulated with LPS for 24 h, were pulsed with OVA_323–339_ peptide (0.1 µg/ml) for 4 h, and were injected subcutaneously (*s.c.*) into foot pads of pretreated mice (5×10^4^ BM-DCs per foot pad). Starting on the day of injection, foot pad swelling was measured daily.

### In Vivo Killing Assay

Spleen cells derived from OT-IxLy-5.1 mice were resuspended in PBS (5×10^7^/ml) and injected (200 µl *i.v.*) into mice via the tail vein. Two days later, groups of mice were immunized with OVA (4 µg/mouse), LPS (4 µg/mouse), and DEX particle-based nanovaccines (200 µl per mouse) as indicated. After 5 days, spleen cells were isolated from Ly-5.1 mice. One fraction was pulsed with 1 µg/ml OVA_257–264_ peptide (1 h, 37°C) to serve as the target cell population. Target cells were labeled at a low concentration of CFSE (0.5 µM, CFSE^high^ cells). The other fraction was left unpulsed and was labeled at higher CFSE concentration (0.05 µM, CFSE^low^ cells) to serve as an internal control. Equal numbers of cells from both populations were mixed, and a total of 10^7^ cells in 200 µl of PBS was injected *i.v.* per mouse. Four h after injection, splenocytes were derived from treated mice, and the frequencies of CFSE^+^ Ly-5.1^+^ cells were assessed by FACS analysis to determine the extent of *in vivo* killing. The level of specific cytotoxicity was calculated according to the following formula: 100%-(CFSE_low_/CFSE_high_x100%).

### Antibody Detection

Mice were immunized with DEX particle formulations equivalent to 4 µg of OVA protein (DEX[OVA], DEX[OVA+LPS]) or 200 µl of DEX particles (DEX[−], DEX[LPS]) as indicated. One and two weeks after immunization mice were bled from the retro-orbital plexus. OVA-specific IgG1 and IgG2a levels were determined in derived sera by ELISA. The antibody titer was defined as the reciprocal serum dilution yielding an absorbance reading of OD = 0.2 after linear regression analysis. IgG contents were standardized by testing reference sera in parallel.

## Results

### Characterization of Functionalized DEX Particle Formulations

**Figure 1:**
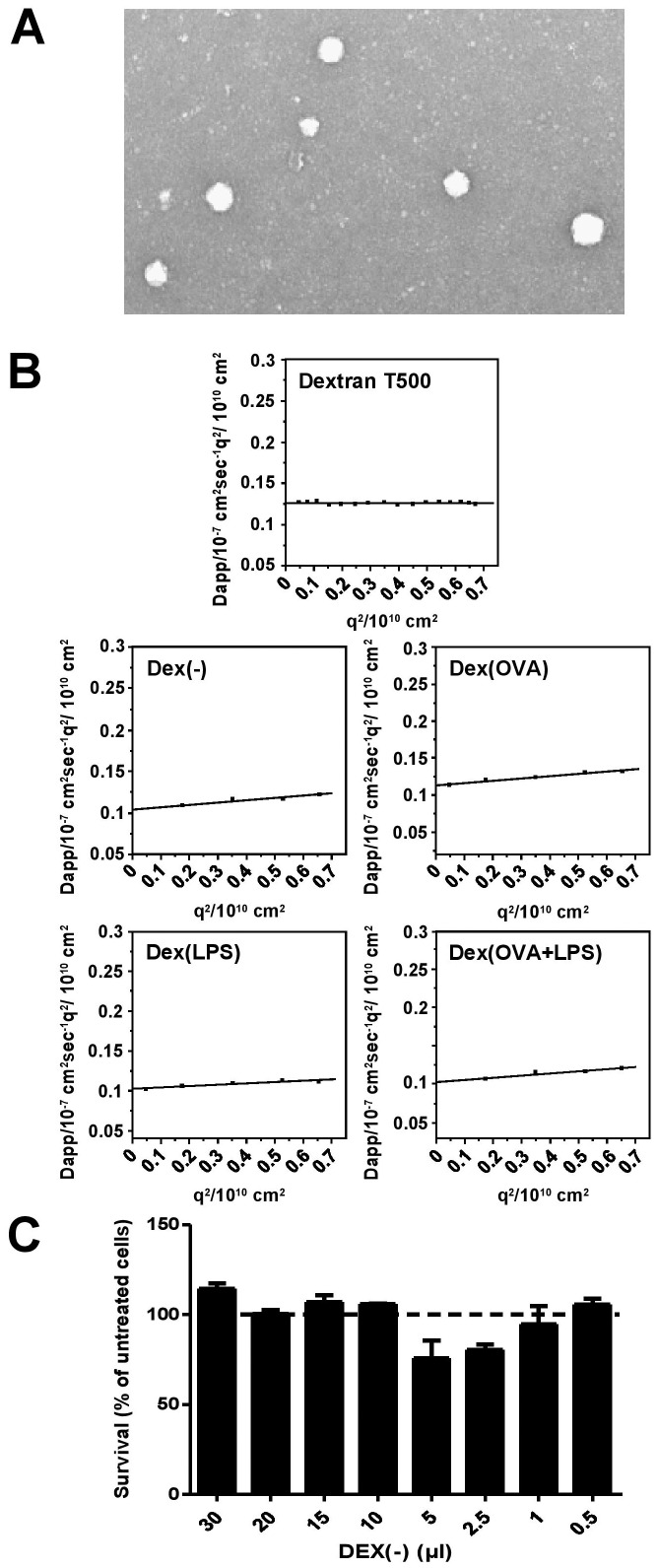
DEX particles are of spherical appearance and uniform in size. (A) Shape and size distribution of DEX(−) dispersed in PBS was studied by electron microscopy. (B) Hydrodynamic radii of Dextran T500 and derived DEX particle formulations as function of q^2^ in DPBS buffer (0.33 mg/ml) were determined by DLS (see Methods). Graphs denote the angular dependency of the apperent diffusion coefficient of the different dextran solutions in buffer solution. (C) To assess cytotoxic effects of DEX particles on BM-DC viability, cells (2.5×10^5^) were seeded into wells of 96 well cell culture plates in a volume of 50 µl in triplicates, and DEX(−) particles (20 mg/ml) were added at different amounts as indicated. One day later, viability of BM-DCs was assessed as described (see Methods). The viability of untreated BM-DCs was set to 100% (dashed line).

DEX-based nanoparticles (DEX[−]) were of spherical shape and rather uniform in size as assessed by electron microscopy (Figure 1A). DEX particle formulations containing OVA and LPS either alone or in combination were comparable in terms of appearance and size (data not shown). Actual sizes of dextran T500 and the different derived types of DEX-based particles were assessed by DLS analysis. The angular dependency of the hydrodynamic radii of the different types of dextran particles is shown in Figure 1B. Extrapolation to zero scattering angle (scattering vector q = 0) resulted in the z-average values of the hydrodynamic radii of the untreated Dextran T500 polysaccharide (<R_h_^−1^>_z_^−1^ = 17 nm), which were somewhat larger in case of the DEX particle formulations (DEX[−]: 23 nm, DEX[OVA]: 19 nm, DEX[LPS]: 19 nm, and DEX[OVA+LPS]: 20 nm). Due to the largely comparable sizes and scattering intensities of Dextran T500 and derived DEX particles, the latter most likely consist of single dextran molecules interacting with OVA and LPS, respectively, in a yet unknown manner, not elucidated in the present work. DLS analysis of different DEX particle types (DEX[OVA], DEX[LPS]) preincubated with human serum showed no significant alterations of particle-associated parameters (Figure S1), which excludes considerable interaction of these DEX particles with serum components.

### OVA-containing DEX Nanoparticles are Engulfed by BM-DCs in a Mannose Receptor-dependent Manner

**Figure 2:**
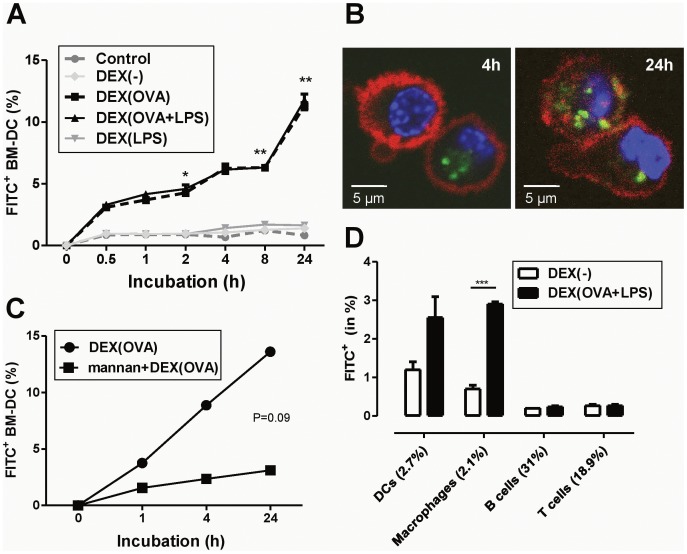
BM-DCs engulf DEX particle formulations in an OVA-dependent manner, mainly via the MR. Aliquots of unstimulated day 6 BM-DCs (5×10^5^ cells; C57BL/6) were left untreated (−) or were coincubated with FITC-labeled DEX particles in duplicates as indicated (each 50 µl). (A) Aliquots were removed after the indicated period of time, and the frequencies of FITC^+^CD11c^+^ BM-DCs were assessed by flow cytometry. Data represent mean±SEM of duplicates and are representative of three independent experiments. Statistical significant differences between OVA-containing DEX particles (DEX[OVA], DEX[OVA+LPS]) and the corresponding control group (DEX[−], DEX[LPS]) are indicated for each time point (*p<0.05, **p<0.01). (B) Cellular uptake of FITC-labeled DEX(OVA) by BM-DCs, stained with anti-CD11c antibody (red) and DAPI (blue), was assessed by confocal laser scanning microscopy 4 h (left panel) and 24 h (right panel) after the onset of coincubation. (C) In parallel cultures, aliquots of BM-DCs were left untreated or were incubated with mannan at high dose (200 µg/ml) for 30 min. Afterwards, DEX(OVA + LPS) was added to either group. Aliqouts of BM-DCs were harvested at the indicated time points, stained for CD11c, and analyzed by flow cytometry. Data represent mean±SEM of duplicates and are representative of three independent experiments. (D) Spleen cells derived from C57BL/6 mice (2×10^6^ cells/200 µl) were left untreated (data not shown) or were coincubated with FITC-labeled DEX particles as indicated (each 30 µl) for 24 h. Afterwards, the cells were stained with either of the indicated cell lineage markers (DCs: CD11c-PE-Cy7, Macrophages: F4/80-eFlour405, B cells: CD19-APC, T cells: CD3-PE), and were analyzed by flow cytometry for the frequency of FITC^+^ cells within either lineage. Numbers in brackets indicate the overall frequency of either cell lineage within the spleen cell suspension. Data represent mean±SEM of three independent experiments. Statistical significant differences between groups are indicated (***p<0.001).

Since DEX-based particles exerted no detrimental effect on BM-DC viability (Figure 1C), next we assessed the intracellular uptake of FITC-labeled DEX particles by unstimulated BM-DCs. In a time kinetics assay DEX particle types devoid of OVA protein (DEX[−], DEX[LPS]) showed no binding to BM-DCs over 24 h of coincubation (Figure 2A). In contrast, incubation with OVA-containing DEX particle formulations (DEX[OVA], DEX[OVA + LPS]) resulted in steadily increasing frequencies of FITC^+^ BM-DCs. Confocal microscopy confirmed pronounced cellular uptake of OVA-containing versus non-containing DEX particles by BM-DCs (Figure 2B) as assessed 4 h (left panel) and 24 h (right panel) after the onset of coincubation. In light of the OVA-dependent binding and uptake of DEX particles by BM-DCs, we asked for involvement of the MR receptor. In competition experiments, preincubation of BM-DCs with the prototypic MR ligand mannan at high concentration significantly reduced cellular binding of subsequently applied DEX(OVA) (Figure 2C).

Based on the finding of MR-dependent binding of DEX particles to BM-DCs, we evaluated their suitablility to specifically target primary APCs as well, an important prerequisite for their intended *in vivo* application. For this, isolated spleen cells were coincubated with FITC-labeled DEX particles. As shown in Figure 2D, only CD11c^+^ DCs and F4/80^+^ macrophages efficiently bound FITC^+^ DEX(OVA+LPS), but not control DEX particles. In contrast, CD19^+^ B cells and CD3^+^ T cells, known to lack MR expression, showed no efficient binding of either type of DEX particles.

### LPS-loaded DEX Formulations Efficiently Stimulate BM-DCs, and DEX Particles Codelivering OVA and LPS Evoke Strong CD4^+^ T Cell Proliferation

In previous studies, polymers functionalized with mannose to target APCs via binding to the MR mediated efficient internalization, but at the same time induced DC activation [12]. Therefore, we analyzed the different types of DEX particles for their DC-activating capacity.

**Figure 3:**
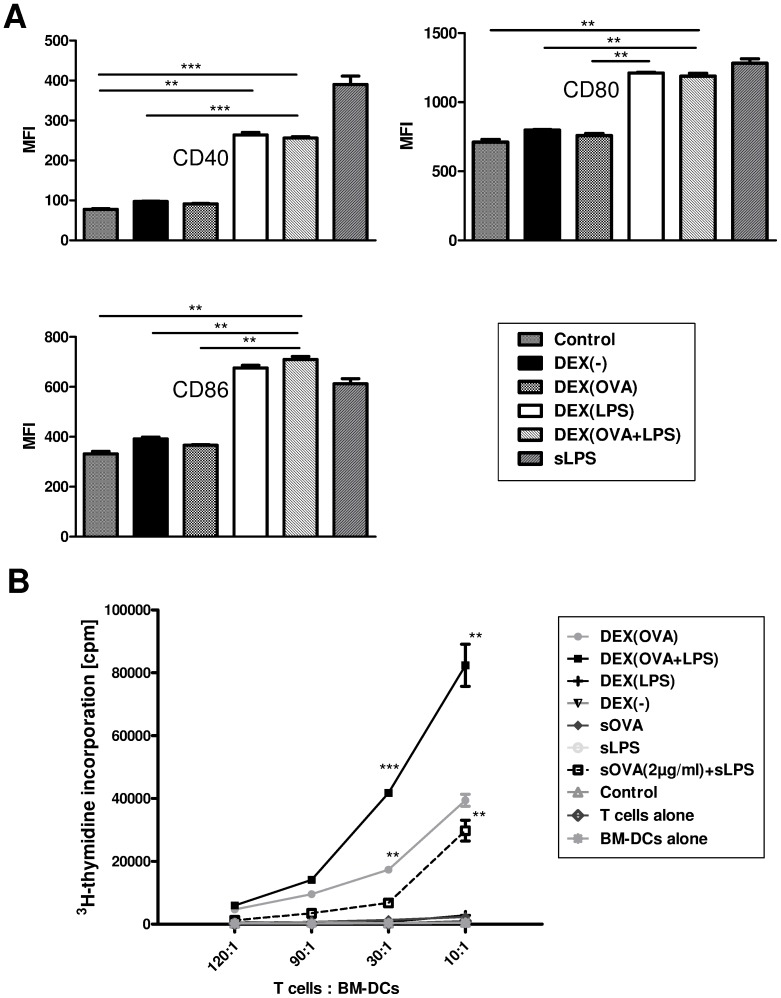
BM-DCs are strongly activated by LPS-containing DEX particle formulations, and codelivery of OVA results in robust antigen-specific CD4^+^ T cell activation. (A) Aliquots of unstimulated day 6 BM-DCs (10^6^ cells; C57BL/6) were left untreated (−), stimulated with LPS (100 ng/ml), or were coincubated with DEX particles (100 µl) as indicated for 24 h. Afterwards, expression of CD40, CD80, and CD86 was assessed by flow cytometry. Graphs denote mean fluorescence intensities (MFI) ± SEM of three experiments. Statistical significant differences between groups are indicated (**p<0.01, ***p<0.001). (B) Unstimulated day 6 BM-DCs (10^6^ cells; C57BL/6) were treated with soluble OVA (2 µg) or as described in *A* for 24 h. Titrated numbers of BM-DCs were cocultured with sorted CD4^+^ OT-II T cells in triplicates for 3 days at the ratios indicated. T cell proliferation was assessed as incorporation of ^3^H-thymidine added for the last 16–18 h. Data represent mean±SEM of triplicates and are representative of three independent experiments. Statistical significant differences between DEX(OVA-LPS) versus OVA plus LPS and DEX(OVA) versus OVA for each T cell/BM-DC ratio are indicated (*p<0.05, **p<0.01).

For this, unstimulated BM-DCs were incubated in parallel assays with OVA protein, LPS, or the different types of DEX particles for 24 h, and the expression of DC activation markers (CD40, CD80, CD86) was analyzed. Stimulation of BM-DCs with LPS resulted in marked upregulation of either activation marker, while OVA and OVA-containing DEX particle formulations (DEX[OVA]) were devoid of DC-stimulatory activity (Figure 3A). Only DEX particle types containing LPS (DEX[LPS], DEX[OVA + LPS]) facilitated robust DC activation as reflected by upregulation of CD40, CD80 and CD86, to similar extent as mediated by LPS in case of the two latter.

Due to efficient engulfment of OVA-adsorbed DEX particles and the DC-stimulatory capacity of particulate LPS, we tested the efficacy of DEX particles to mediate an antigen-specific T cell response. While BM-DCs preincubated with soluble OVA protein alone induced no marked proliferation of subsequently cocultured OVA peptide-specific OT-II CD4^+^ T cells, pretreatment of BM-DCs with DEX(OVA) facilitated robust T cell activation (Figure 3B). In accordance, pretretment of BM-DCs with DEX particles codelivering OVA and LPS (DEX[OVA+LPS]) facilitated strongly enhanced T cell activation, which was significantly higher as induced by BM-DCs pretreated with OVA plus LPS.

### Codelivery of OVA and LPS by a DEX-based Nanovaccine Results in a Potent and Sustained Immune Response *in vivo*

**Figure 4:**
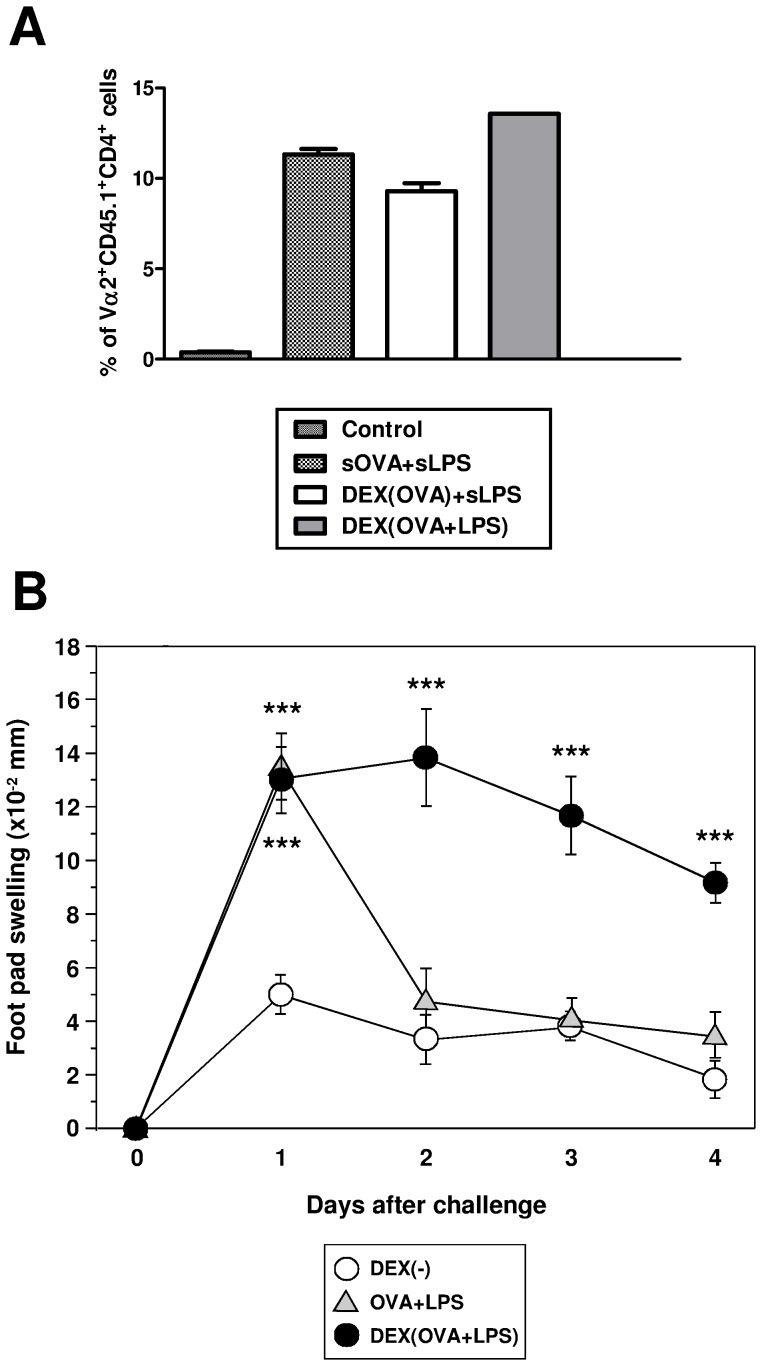
DEX particles containing OVA and LPS induce strong activation of CD4^+^ antigen-specific T cells when applied *in vivo*. (A) Mice (C57BL/6) received CFSE-labeled OVA-specific OT-II T cells (10^7^/mouse) *i.v*. Two days later, mice (three animals per group) were immunized *i.v.* with OVA (4 µg per mouse), LPS (0.4 µg), and DEX particles (each 200 µl) as indicated. After another three days, frequencies of CD4^+^CD45.1^+^Vα2^+^ OT-II T cells in spleen cell suspensions were analyzed by flow cytometry. Data represent mean±SEM of two independent experiments. The frequencies of proliferating CD4^+^ OT-II T cells in either treated group were signifiantly higher than in the non-immunized control group. (B) CD4^+^ DO11.10 T cells (5×10^6^) were transferred to BALB/c mice (3 animals per group). One day later, OVA_323–339_ peptide (40 µg), LPS (100 ng), or different DEX particle formulations (each 40 µg) were injected as indicated. For challenge, LPS-stimulated BM-DCs were pulsed with OVA_323–339_ peptide (0.1 µg/ml), and were injected into either hind foot pad of pretreated BALB/c mice. Food pad thickness was recorded daily. Data represent mean±SEM of six recordings per group. Statistical significant differences between any group versus the control group (DEX[−]) are indicated (*p<0.05, **p<0.01, ***p<0.001).

Due to the strong bioactivity of OVA and LPS when applied as particulate formulations to mediate DC-dependent activation of antigen-specific CD4^+^ T cells *in vitro*, we assessed the suitability of these DEX particles to mount an OVA-specific CD4^+^ T cell response, when applied directly *in vivo*, which requires targeting of MR-expressing APCs. To this end, proliferation of CFSE-labeled splenocytes derived from OT-II mice and injected *i.v.* into C57BL/6 mice was analyzed after treatment of recipient mice with OVA and LPS as soluble or particulate formulations. As shown in Figure 4A, in all groups of mice which had received OVA plus LPS, strong proliferation of OT-II T cells was detected.

Based on this result, the suitability of DEX-based nanovaccines to elicit a robust CD4^+^ T cell-dependent immune response was evaluated in a model of antigen-specific foot pad swelling. For this, syngeneic OVA peptide-specific CD4^+^ T cells were transferred into recipient mice, sensitized with OVA plus LPS, DEX(OVA+LPS) or DEX(−) as a control, and challenged by injection of syngeneic, OVA peptide-pulsed stimulated BM-DCs into the hind foot pads. While sensitization with soluble OVA plus soluble LPS resulted in a transient foot pad swelling only, that returned to background levels already at day 2 after challenge, application of particle-bound OVA plus LPS (DEX[OVA+LPS]) induced a marked and prolonged delayed-type hypersensitivity response that persisted for days (Figure 4B).

### DEX-based Nanovaccines Induce Strong CD8^+^ T Cell Activation *in vivo*

**Figure 5:**
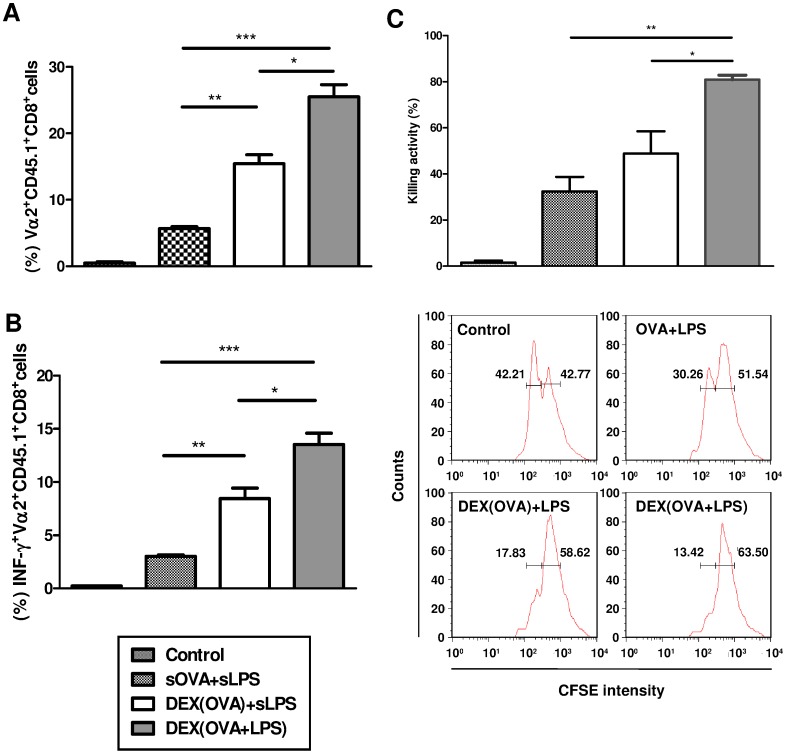
The nanovaccine DEX(OVA+LPS) induces profound activation of antigen-specific CD8^+^ T cells *in vivo.* C57BL/6 mice received CFSE-labeled, OVA-specific OT-I T cells (10^7^) *i.v.* Two days later, groups of mice (each five animals) were either left untreated or were immunized with OVA (4 µg per mouse), LPS (4 µg), and DEX particle formulations (each 200 µl) as indicated. (A) On day 5, the frequencies of proliferating CD8^+^CD45.1^+^Vα2^+^ OT-I cells were determined in spleen cell suspensions by flow cytometry. (B) In the same experiments, the frequencies of IFNγ^+^ OT-I T cells were assessed by flow cytometry. (A,B) Data represent mean±SEM of two independent experiments each. The frequencies of proliferating and IFN-γ producing CD8^+^ OT-I T cells in either group were signifiantly higher than in the non-immunized control group. Other statistical significant differences between groups are indicated (*p<0.05, **p<0.01, ***p<0.001). (C) On day 4 after immunization, mice were injected with CFSE^low^ target cells (loaded with OVA_257–264_) and CFSE^high^ control cells (each 10^7^ cells) derived from syngeneic Ly-5.1^+^ mice. 4 h later, splenocytes were isolated and frequencies of CFSE-labeled cell populations were assessed by flow cytometry. Upper panel: Data represent mean±SEM of two independent experiments. Statistical significant differences between groups are indicated (*p<0.05, **p<0.01). Lower panel: Frequencies of Ly-5.1^+^ target cells (CFSE^low^) and control cells (CFSE^high^) in spleen cell suspensions derived from one mouse of either group are shown as histograms. Graphs are representative of two independent experiments.

To assess the suitability of DEX-based nanovaccines to induce robust CD8^+^ T cell responses, the proliferation of OVA peptide-specific CD8^+^ T cells derived from OT-I mice and transferred into syngeneic C57BL/6 mice was assessed after treatment of recipient mice with OVA and LPS in different formulations. In comparison, coapplication of soluble OVA and LPS mounted low CD8^+^ T cell proliferation only, which was significantly higher in case of prior immunization with particulate OVA (DEX[OVA]), coadministered with soluble LPS (Figure 5A). However, DEX particles adsorbed with OVA plus LPS (DEX[OVA+LPS]) evoked the strongest OVA-specific CD8^+^ T cell proliferation of all groups compared. Similarly, the frequency of OT-I T cells producing the Th1 cytokine IFN-γ was lowest in mice treated with OVA plus LPS, intermediate when DEX(OVA) plus LPS had been coapplied, and highest in the group immunized with DEX(OVA+LPS) (Figure 5B).

The finding of robust CD8^+^ T cell proliferation and IFN-γ production as induced by DEX-based nanovaccines *in vivo* prompted us to assess the functional activity of OT-I T cells in terms of cytotoxic activity. In an *in vivo* killing assay, lysis of OVA peptide-presenting target cells occurred only in groups of mice cotreated with OVA and LPS (Figure 5C, upper panel). Lysis of CFSE^low^ target cells was lowest after coadministration of soluble OVA and LPS, somewhat elevated in the group which had received DEX(OVA) plus LPS, and strongest in mice injected with DEX(OVA+LPS) (Figure 5C, lower panel).

### DEX Particles that Codeliver OVA and LPS Induce a Th2-biased Humoral Response

**Figure 6:**
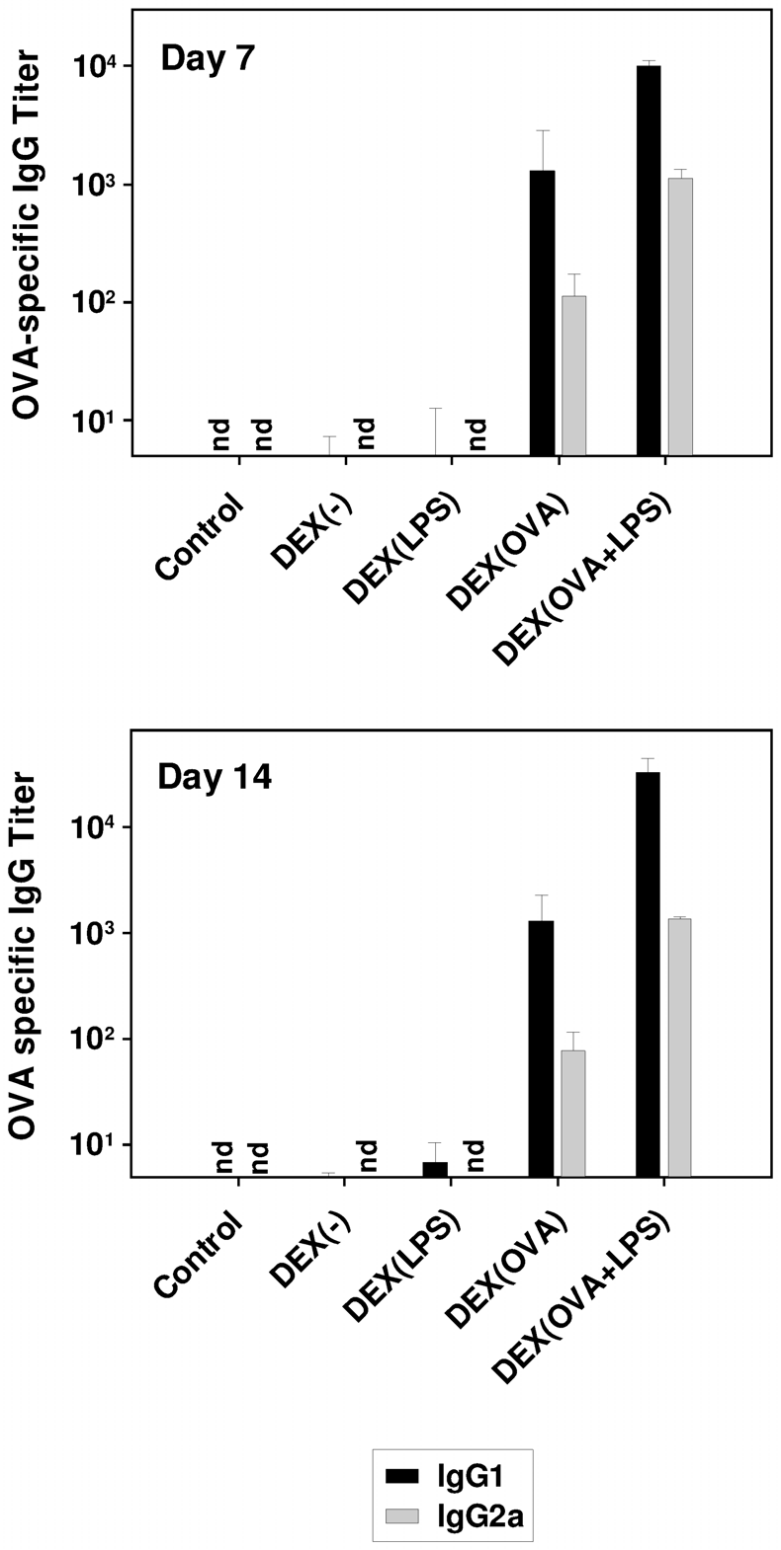
DEX particles containing OVA elicit a specific Th2-biased humoral immune response, augmented upon codelivery of LPS. Naive C57BL/6 mice (two mice per group) were immunized with DEX particle formulations as indicated. On days 7 (upper panel) and 14 (lower panel), mice were bled and derived sera were used for detection of OVA-specific IgG1 and IgG2a antibody titers. Data represent mean±SEM of two sera per group.

In light of the essential role of humoral immune responses for pathogen clearance and their contribution to anti-tumor responses, we asked for the potential of DEX-based nanovaccines to mount the production of OVA-specific antibodies. For this, naive mice were injected *i.v.* with the different types of DEX particles, and sera derived one and two weeks later were assayed for OVA-specific IgG titers. At either time point, OVA-specific IgG1 and IgG2a were detectable only in sera obtained from mice immunized with OVA-containing DEX-based nanovaccines, thereby confirming antigen-dependency of antibody production (Figure 6). As expected, OVA-specific antibody titers were higher when OVA plus LPS were codelivered (DEX[OVA+LPS]) than mounted in response to OVA alone. At either time point assessed, more IgG1 than IgG2a was detected, reminiscent of a Th2-skewed IgG pattern. Taken together, these findings show that DEX-based nanovaccines are capable to induce both a cellular and a humoral immune response *in vivo* in an antigen-specific manner.

## Discussion

DCs constitute an attractive target for immunotherapeutic strategies based on their versatile functional properties, namely to maintain peripheral tolerance under steady state conditions [13], but to induce potent immune responses when activated by pathogen-associated danger signals [14]. In conventional vaccination strategies protein antigen(s) and APC-activating adjuvant(s) are coinjected [15]. However, it is well established by now, that antigen in combination with adjuvant induces a stronger immune response when codelivered as a particulate formulation [16].

Ongoing studies aim on the development of reliable targeting strategies which enable APC-focussed delivery of nanovaccines [17], but major restrictions arise from the laborious synthesis and functionalization, limited biodegradability, cytotoxicity, and intrinsic immunomodulatory properties of such formulations [18]. In light of these limitations, we sought to develop a nanovaccine largely devoid of the aformentioned hindrances.

For this, we evaluated the suitability of DEX particles which have been introduced almost thirty years ago as a biodegradable, and non-cytotoxic carrier system for proteins and other compounds, reported to elicit antigen-specific humoral responses *in vivo* after *s.c.* immunization of mice, at much higher extent than direct immunization with antigen [4].

In accordance with the general properties of dextran biopolymers, in our study DEX particles were devoid of cytotoxic or direct immunomodulatory effects. In this regard, it is noteworthy that other types of nanoparticles, like zinc oxide [19] or amorphous silica nanoparticles [20], which are contained in a variety of consumer products, have been shown to induce cytotoxic effects in isolated immune cells.

In earlier approaches, particulate carriers have been optimized in terms of structural composition and size, aimed to result in enhanced uptake by myeloid APCs by means of endocytosis and phagocytosis [21]. Based on these studies, particles of 0.1–1 µm in diameter have been demonstrated to passively target APCs [22], [23]. However, cell-type unspecific uptake of such types of particles may limit their usability in terms of APC-specific vaccination [24]. In contrast, here we show that DEX particles as such, despite their minor size, were not engulfed by immune cells to a great extent, which underlines their potential to serve as inert carriers for macromolecules that facilitate cell type-specific targeting.

Accordingly, we sought to exploit the intrinsic myeloid APC targeting property of OVA, which by itself constitutes an important model antigen frequently employed to study adaptive immune responses. In agreement with the well established MR-targeting properties of OVA [6], [7], here we demonstrated that DEX particles containing OVA were efficiently engulfed by murine BM-DCs in an MR-dependent manner, as suggested by efficient inhibition in the presence of mannan at high concentration. Moreover, OVA-containing DEX particles efficiently bound primary DCs and macrophages, shown to express the MR [25], while B cells and T cells as MR-deficient lymphoid immune cells were not targeted. These results suggest that mannosylation of a given protein antigen may suffice to mediate binding and cellular uptake of a conjugated nanovaccine by MR-expressing APCs. Accordingly, as exploited in our study, a candidate protein at the same time may serve both as a source of antigen, and as an APC-targeting molecule. In confirmation of the latter, the uptake of protein antigens by APCs was strongly elevated when these proteins were mannosylated due to expression in engineered yeast cells [26] or *in vitro* prior to application [27].

Several studies have suggested that uptake of MR-targeting nano-carriers, like polyanhydride [12] and PLGA (D, L-lactide-co-glycolic acid) [28] nanoparticles, resulted in DC activation, which may be explained in part by signaling pathways activated in response to MR-mediated protein uptake [29]. In contrast, in our study BM-DCs incubated with DEX(OVA) remained unstimulated, which indicates that MR engagement as such is not sufficient to mediate DC activation. In general, structurally distinct nanoparticle formulations were characterized by an intrinsic DC-activating immunomodulatory function, i.e. γ-PGA (poly[γ-glutamic acid] [30], poly(propylene)sulfide [31], or LDH (layered double hydroxide) [32]. Yet other types of nanoparticles have been reported to exert inhibitory activity on DCs, like PVA-SPIONS (poly[vinylalcohol]-coated super-paramagnetic iron oxide nanoparticles) [33]. Altogether, these reports demonstrate that nanoparticles often exert immunomodulatory activity, which may modulate the character of an intentionally induced immune response in an unwanted manner, e.g. in terms of T cell polarization [34]. Therefore, the lack of immunomodulatory activity of DEX particles on DCs clearly broadens their range of application, because it may allow to shape a nanovaccine-induced immune response solely according to the properties of codelivered adjuvants [35]. Here we employed the TLR4 ligand LPS as an adjuvant, well known to activate myeloid DCs, which in turn favor Th1-biased immune responses [36]. LPS-containing DEX particles readily activated BM-DCs to similar extent as LPS applied directly. Interestingly, DEX particles engineered to contain only LPS were not internalized by BM-DCs. In contrast, Demento and co-workers [37] reported that PLGA-based nanoparticles were engulfed by murine DCs at higher efficiency when decorated with LPS than at non-functionalized state. This observation suggests that TLR4 engagement *per se* may be sufficient for subsequent internalization of TLR4 ligand-coated nanovaccines. However, the discrepancy between the findings of Demento and co-workers and our results may be explained by differences in particle-surface LPS densities.

The DEX-based nanovaccine which codelivered OVA and LPS (DEX[OVA+LPS]) was most effective in inducing pronounced T cell responses both *in vitro* when incubated with BM-DCs, as well as *in vivo* after direct application. Whereas both soluble and DEX-bound OVA were able to stimulate the proliferation of CD4^+^ T cells, only the delivery of DEX(OVA+LPS) exerted a sustained immune response as demonstrated in a foot pad swelling assay, a well established model for T cell mediated delayed-type hypersensitivity. DEX-based nanovaccines also proved substantially more efficient to mediate cross presentation of OVA peptides by DCs *in vivo* than soluble OVA [5] as evidenced by the induction of a Th1-biased activation of OVA-specific CD8^+^ OT-I T cells. Taken together, these data demonstrate that the immobilization of antigen (OVA) and adjuvant (LPS) on a particulate carrier (DEX) that targets DCs induces superior T cell-mediated immune responses *in vivo* when compared to immunization with soluble antigen and adjuvant.

Besides mounting potent T cell responses, in line with the results obtained by Schröder et al. [4], DEX-based nanovaccines containing OVA also induced a Th2-biased OVA-specific IgG isotype pattern (IgG1>IgG2a). Thus, in context with DEX particle-derived nanovaccines other adjuvants than LPS may be required to induce a pronounced Th1-skewed pattern of antibody production.

Taken together, the modular character of the DEX-based nanovaccine platform evaluated in this study may enable the generation of vaccine formulations that are able to specifically target glycosylated protein antigens to DCs in a MR-mediated fashion *in vivo*. In addition, these DEX-based nanocarriers are also able to specifically administer adjuvants or other immunomodulatory agents to DCs *in situ* in order to shape immune responses as required for immunotherapeutic applications.

## Supporting Information

Figure S1 **DEX particles functionalized with OVA or LPS display no interaction with serum.** DLS analysis of DEX particle formulations preincubated with human serum was performed as described in the Materials and Methods section. Graphs denote correlation functions (scattering angle 30°) of DEX(OVA) (upper panel) and DEX(LPS) (lower panel) in human serum. Force fit (eq. 3 and residuum (bottom line) are shown.
(TIF)

Methods S1 **Detailed information on the generation of DEX particles, and of DLS analysis are given in Methods S1.**
(DOC)
