# Supplementary figures and images for: A Trifunctional Dextran-Based Nanovaccine Targets and Activates Murine Dendritic Cells, and Induces Potent Cellular and Humoral Immune Responses In Vivo
Source: PLoS One. 2013 Dec 5;8(12):e80904. doi: 10.1371/journal.pone.0080904 (PMC3855172; doi:10.1371/journal.pone.0080904)

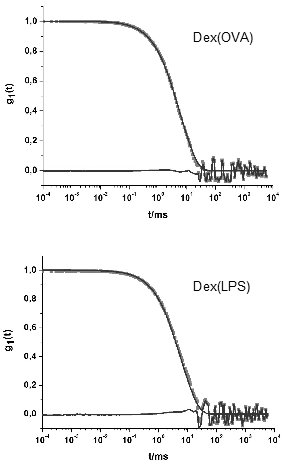

Supplement: Figure S1 — DEX particles functionalized with OVA or LPS display no interaction with serum. DLS analysis of DEX particle formulations preincubated with human serum was performed as described in the Materials and Methods section. Graphs denote correlation functions (scattering angle 30°) of DEX(OVA) (upper panel) and DEX(LPS) (lower panel) in human serum. Force fit (eq. 3 and residuum (bottom line) are shown. (TIF) [file pone.0080904.s001.tif]
