# Supplementary material for: A Trifunctional Dextran-Based Nanovaccine Targets and Activates Murine Dendritic Cells, and Induces Potent Cellular and Humoral Immune Responses In Vivo
Source: PLoS One. 2013 Dec 5;8(12):e80904. doi: 10.1371/journal.pone.0080904 (PMC3855172; doi:10.1371/journal.pone.0080904)
Supplement: Methods S1 — Detailed information on the generation of DEX particles, and of DLS analysis are given in Methods S1. (DOC) [file pone.0080904.s002.doc]

**Methods S1**

Functionalization of dextran (DEX) particles with antigen and adjuvant

To generate DEX-based nanoparticles, 7.69 g Dextran T500 with an average Mw of 500 kDa (Pharmacosmos A/S, Holbaek, Denmark) was dissolved in distilled water (26% w/v). In some preparations, 20 mg of chicken ovalbumine (OVA) protein (Sigma-Aldrich, Deisenhofen, Germany), and 125 µg lipopolysaccharide (LPS, Sigma-Aldrich) were dissolved in 400 µl of PBS, and were subsequently added to the dextran solution, respectively. Then, 30 ml of customary vegetable oil pre-cooled to 4°C was added, and the mixture was emulsified by magnetic stirring for 20 min at 4 °C, followed by sonification (BM70; Bandelin electronic, Berlin, Germany) in an ice-cooled water bath for 40 s. The resulting emulsion was slowly poured into 200 ml of 0.1 % (w/v) solution of Tween 80 (STRVA, Feinbiochemica, Heidelberg/New York) in acetone under constant stirring to precipitate DEX nanoparticles at room temperature. After 6 h, DEX particles were filtered through a cell strainer (40 µm diameter) to remove aggregates. Derived DEX particles were washed 3 times with 0.1 % Tween 80 in acetone solution and collected by centrifugation at 3,000 g for 5 min. DEX particles were resuspended in 2 ml of 1% (w/v) Tween 80/acetone solution and air dried at room temperature. In some preparations, FITC-conjugated dextran T500 (Sigma-Aldrich) was used.

DEX particles were resuspended in PBS and were stored at 4°C for up to several months for subsequent analysis. To determine concentrations of DEX-bound OVA protein, particles were disrupted by ultrasonication. Concentrations of released OVA were measured by applying the BCA^TM^ protein assay kit (Pierce, Rockford, IL). By this assay, OVA-containing DEX particles were shown to contain about 200 µg of OVA/µl of undiluted particles. The LPS content of functionalized particles was determined using the ToxinSensor^TM^ chromogenic LAL endotoxin assay kit as recommended by the manufacturer (GenScript, NJ). By this assay DEX(LPS) was shown to contain about 13 pg of surface accessible LPS per µl of undiluted DEX particles, while control DEX particles (DEX[-]) were devoid of LPS. Supernatants of precipitated DEX(LPS) and DEX(OVA+LPS) removed several weeks to months after particle preparation exerted no effect on BM-DC surface marker expression, thereby confirming tight binding of LPS (data not shown).

Dynamic light scattering (DLS)

Solutions of DEX particles for light scattering experiments were prepared in a dust free flow box. Cylindrical quartz cuvettes (20 mm diameter, Hellma, Müllheim, Germany) were cleaned with dust-free destilled acetone. DEX particle solutions were prepared in DPBS buffer (Life Technologies, Darmstadt, Germany) at a concentration of 0.33 mg/ml and filtered through Milex AA filters with 800 nm pore size (Millipore, Billerica, MA) before use. DLS experiments were performed with an apparatus consisting of a HeNe laser (632.8 nm, 25 mW output power), an ALV-CGS 8F SLS/DLS 5022F goniometer equipped with eight simultaneously working ALV 7004 correlators, and eight QEAPD Avalanche photodiode detectors. Correlation functions were fitted by a sum of two exponential functions, from which the first cumulant (initial slope versus correlation time t) was calculated resulting in an angular-dependent diffusion coefficient *D_app_(q),* q being the scattering vector (q = 4πn sin(θ/2)/λ_o_, with n the refractive index of the solvent, θ the scattering angle and λ_o_ the wavelength of the light source*.* By extrapolation of *D_app_(q)* to *q = 0* the z-average of the diffusion coefficient, *D_z_*, is obtained, which yields the hydrodynamic radius R_h_  <R_h_^-1^>_z_^-1^ by formal application of Stokes law.

The aggregation behavior of DEX particles in human serum was investigated by DLS [10]. For these measurements, 1 ml human serum (c(serum=59g/L) and 1 ml particles (c=0.01 g/l) dissolved in DPBS buffer solution (Life Technologies) were subsequently added to the light scattering cuvette. Then, the cuvettes were incubated for 20 minutes on a shaker at room temperature before measurement.

Data evaluation was performed as reported before [10]. Briefly, the obtained correlation functions of the DEX particles and human serum can be perfectly described by a sum of two (eq. 1) and three exponential functions (eq. 2), respectively. The following fit functions were applied:

Dextran (*np*):
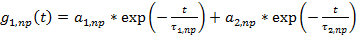
 (1)

Serum (*s*):
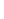

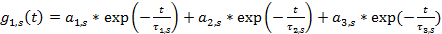
 (2)

In the equations above a_i_ represents the amplitudes of the exponential functions with decay times τ_i_ = 1/(q^2^D_i_), with D_i_ the Brownian diffusion coefficient of component i. If no or negligible particle-serum interaction exists, the correlation function of the serum particle mixture, *g_1,m_(t)*, should be perfectly fitted by the sum of the individual correlation functions, *g_1,np_(t)* and *g_1,s_(t)* according to


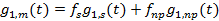
 (3)

with the amplitudes *f_s_* and *f_np_* the only fit parameters. Serum-induced nanoparticle aggregation would be sensitively detected by significant systematic residues between the measured and the fitted correlation functions according to eq. 3.
